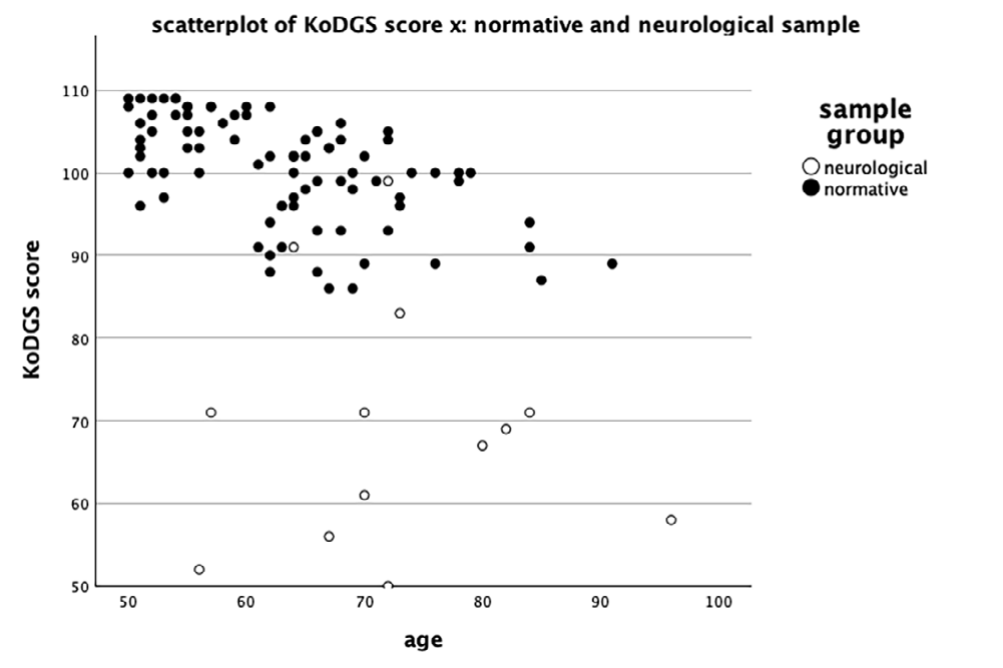# A neurocognitive screening tool for deaf sign language users in Germany: the KoDGS

**DOI:** 10.1002/alz70858_097147

**Published:** 2025-12-24

**Authors:** Lisa M. Stockleben, Bencie Woll, Joanna Atkinson

**Affiliations:** ^1^ University of Cologne, Cologne, North‐Rine Westphalia, Germany; ^2^ University College London, London, London, United Kingdom

## Abstract

**Background:**

Existing neurocognitive and language assessments provide normative data only for hearing or hard‐of‐hearing people (Brünecke et al., 2018; Völter et al., 2023) with inaccurate diagnosis and inadequate care for sign language using communities. The British Sign Language Cognitive Screening Test (BSL‐CST) (Atkinson et al., 2015) is a sign language‐based, normed, validated and clinically applied cognitive screening tool designed to identify dementia in BSL users. Several projects are currently working on adaptations into other sign languages (Tsatali et al., 2023). This paper presents the German sign language (DGS) adaptation.

**Method:**

A culturally and linguistically appropriate DGS version (the KoDGS) was produced through an extensive adaptation process. The KoDGS assesses orientation, attention, memory, language, semantic verbal fluency, visual‐spatial abilities and perception as well as executive functions. It was administered to 99 deaf individuals (age: 50 ‐ 96 yrs; *M* = 64.33), together with social and neurological histories. Convergent measures were applied to check for test validity.

**Result:**

The KoDGS significantly differentiates between normative (*n* = 86) and neurological (*n* = 13) samples, with a threshold for clinical test application. Positive correlations between convergent measures indicate robust test validity.

**Conclusion:**

This paper presents the first cognitive screening tool in DGS, with potential to improve neuropsychological screening of signers in Germany. The study makes a significant contribution to research on diagnosing dementia in underserved minority communities, outlining a process for cultural and linguistic adaptation of cognitive screening tools, applicable to other sign languages and deaf communities.

**References**

1. Atkinson, J., et al. (2015). Detecting Cognitive Impairment and Dementia in Deaf People: The British Sign Language Cognitive Screening Test. *Arch Clin Neuropsych*, *30*(7), 649‐711

2. Brünecke, I., Hölsken, S. & Kessler, J. (2018). Der DemTect Eye+Ear ‐ Neues kognitives Screeningverfahren bei schwerhörigen Menschen mit Demenzverdacht. *Zeitschrift für Audiologie*, 57(3), 121.

3. Tsatali, M., et al. (2023). A Cognitive Screening Test for detecting Alzheimer's Disease Dementia in Deaf older adults in Austria and Greece: the De‐Sign Erasmus+ project. 33^rd^ Alzheimer Europe Conference, Helsinki.

4. Völter, C., et al. (2023). Validation of the German Montreal‐Cognitive‐Assessment‐H for hearing‐impaired. *Front Aging Neurosci*, *15*, https://doi.org/10.3389/fnagi.2023.1209385